# Coronary Revascularization after Transcatheter and Surgical Aortic Valve Replacement

**DOI:** 10.3390/jcm12237257

**Published:** 2023-11-23

**Authors:** Davide Gabbieri, Federico Giorgi, Greta Mascheroni, Matteo Chiarabelli, Giuseppe D’Anniballe, Marco Meli, Clorinda Labia, Italo Ghidoni

**Affiliations:** 1 Cardiac Surgery Unit, Department of Medical-Surgical Cardiology, Hesperia Hospital Modena, 41125 Modena, Italy; dgabbieri@yahoo.it (D.G.); gmascheroni@hesperia.it (G.M.); gdanniballe@hesperia.it (G.D.); mmeli@hesperia.it (M.M.); clabia@hesperia.it (C.L.); italoghidoni@gmail.com (I.G.); 2 Cardiac Surgery Unit, Department of Surgical, Medical and Molecular Pathology and Critical Care, Azienda Ospedaliero Universitaria Pisana, University of Pisa, 56126 Pisa, Italy; 3 Edwards Lifesciences, 20141 Milano, Italy; matteo_chiarabelli@edwards.com

**Keywords:** TAVI, SAVR, PCI, coronary re-access

## Abstract

Introduction: Due to the selective criteria and short-term follow-up of previous transcatheter aortic valve implantation (TAVI) trials, the coronary revascularization incidence after TAVI has been difficult to determine. This study investigated the epidemiology of coronary revascularization after surgical aortic valve replacement (SAVR) and TAVI in patients with severe aortic valve stenosis (AS), with and without coronary artery disease (CAD), in a mid-term follow-up, single-center, real-world setting. Methods: Between 2010 to 2020, 1486 patients with AS underwent SAVR or TAVI with balloon-expandable Edwards^®^ transcatheter heart valves (THVs). Using hospital discharge records, we could estimate for each patient resident in Emilia Romagna the rate of ischemic events treated with percutaneous coronary intervention (PCI). A subgroup without CAD was also analyzed. Results: The 5-year overall survival was 78.2%. Freedom from PCI after AVR and TAVI at 5 years was 96.9% and 96.9%, respectively, with previous PCI as a predictor (HR 4.86, 95% CI 2.57–9.21 *p* < 0.001). The freedom from PCI curves were not significantly different. Conclusions: Notwithstanding the aged population, the revascularization incidence was only 2.4%, requiring further evaluation even in younger patients with longer follow-up. Despite the profile frame raise due to the evolution of Edwards^®^ balloon-expandable THVs, PCI or coronarography feasibility were not compromised in our population.

## 1. Introduction

Surgical aortic valve replacement (SAVR) has been the standard of care for aortic valve stenosis (AS) over the past 50 years. However, in recent decades, transcatheter aortic valve implantation (TAVI) has emerged as the treatment of choice for patients with AS, initially in inoperable and high-surgical-risk patients [1,2] and then also in intermediate-risk patients, with a steady increase in the number of performed TAVI procedures in North America and Europe [3,4]. Moreover, recent trials including low-risk patients reported the non-inferiority or even superiority of TAVI versus SAVR [5,6]. In the PARTNER 3 trial, TAVI with Edwards^®^ (Edwards Lifesciences, Irvine, CA, USA) balloon-expandable technology was compared with SAVR among patients with low surgical risk, showing even superiority in terms of composite endpoint of death, stroke and rehospitalization at 1 year [5]. Non-inferiority of TAVI in low-risk patients was also observed in the comparison of self-expanding technology and SAVR in the randomized Evolut Low-Risk trial [6]. Data from the NOTION and PARTNER-2A studies comparing TAVI with SAVR in patients with low or intermediate surgical risk showed no difference in terms of death and disabling stroke at 5- and 6-year follow-up, respectively [7,8]. According to current European and US guidelines for the management of valvular heart disease, transfemoral TAVI and SAVR are both Class-I-recommended for the majority of patients with severe, symptomatic AS [9,10]. The decision is usually made by local heart teams taking into account multiple and complex clinical and anatomical factors.

When expanded toward low-risk patients, who often are younger, the need for percutaneous coronary intervention (PCI) after TAVI may be more frequent, because the incidence of coronary artery disease increases with age. However, due to the selective criteria in previous TAVI trials and the relatively short-term follow-up available, the coronary revascularization incidence after TAVI has been difficult to determine. The incidence of PCI after TAVI may increase in patients with longer life expectancies, with potential implications for most TAVI procedures and transcatheter heart valve (THV) prosthesis choices. Moreover, reports suggested that coronary re-access may be difficult to establish, as a result of the transcatheter valve positioning [11]. The aim of this study was to assess the coronary revascularization incidence and risk factors during mid-term follow-up after SAVR and TAVI, in patients with severe aortic valve stenosis, with and without coronary artery disease (CAD), in a single-center real-world setting over the past decade.

## 2. Materials and Methods

### 2.1. Patient Source and Data Collection

From January 2010 to December 2020, 1486 patients with native severe aortic valve stenosis underwent SAVR or TAVI with balloon-expandable Edwards (Edwards Lifesciences, Irvine, CA, USA) devices in Hesperia Hospital Modena, Italy. Active endocarditis, previous aortic valve surgery (SAVR or TAVI), mild or moderate aortic valve stenosis or isolated aortic valve regurgitation were the exclusion criteria. Since 2010 in Hesperia Hospital, a heart team has been arranged to discuss and select patients with severe aortic valve stenosis. Thus, inoperable or high-risk patients were selected for TAVI, similarly to PARTNER 1A and 1B [1,2]; only in few cases, due to comorbidities or severe frailty, did moderate- or low-risk patients undergo TAVI. Patients’ data about demographics, comorbidities, preoperative status and procedural, in-hospital, postoperative and long-term outcomes were collected.

### 2.2. Preoperative Variables

Data were collected from RERIC Hesperia Hospital. The “Regione Emilia Romagna Interventi Cardiochirurgia Registry” is a prospective regional database collecting preoperative, intraoperative and postoperative data from patients undergoing cardiac surgical procedures in the six regional cardiac surgery departments (academic hospitals: *n* = 2, private hospitals: *n* = 4). Registry management is centralized; every 3 months, the Hesperia Hospital cardiac surgery department is required to dispatch the data to the regional healthcare agency for quality/completeness control and to monitor cardiac surgery results in the Emilia Romagna region. Personal data, such as gender, age and residency region at the procedure time, and clinical data, like remote pathological history, including other cardiovascular risk factors (diabetes mellitus, obesity, hypertension, dyslipidemia and smoking habits) or other extra-cardiac conditions, such as cerebrovascular events, extracardiac arteriopathy, chronic obstructive pulmonary disease (COPD), frailty and chronic kidney disease (CKD), on dialysis or not, were considered.

The preoperative, procedural and postoperative variables are summarized in Table 1, Table 2 and Table 3.

The length of stay, the 30-day mortality and the in-hospital mortality were recorded. Date and cause of death were obtained from the ANPR (Anagrafe Nazionale della Popolazione Residente) database. Follow-up of the patients living in Emilia Romagna was possible due to the adhesion of Hesperia Hospital Modena to the UNIMORE TAVInAVEN study (Sostituzione valvolare aortica per via percutanea TAVI. Area Vasta Emilia Nord), approved by the Ethical Committee of Area Vasta Emilia Nord on 4 January 2019 with protocol AOU 0000184/19. Using the hospital discharge records (SDO), the cardiovascular disease diagnosis codes related to cardiac ischemic disease (410-414) and the cardiovascular procedure codes related to PCI (36.04 Intracoronary artery thrombolytic infusion, 36.06 Insertion of non-drug-eluting coronary artery stent(s), 36.07 Insertion of drug-eluting coronary artery stent(s), 36.09 Other removal of coronary artery obstruction), we could estimate for each patient living in Emilia Romagna the freedom from ischemic events treated with PCI. Furthermore, we evaluated this freedom excluding the population with coronary artery disease (CAD) before the procedure, such as patients with previous PCI, previous coronary artery bypass grafting (CABG) and previous and/or recent myocardial injury. Then, we evaluated the vascular and bleeding complications in patients who underwent PCI prior to TAVI, even though we could not compare their outcomes with untreated patients because our strategy was to perform PCI, within 60 days of TAVI, in all patients with a higher Syntax Score (≥22) before the PCI, or with a higher residual Syntax Score (>8) after a previous PCI.

All cases of PCI after TAVI were analyzed. The risk plane was determined using CT scanning and classified into three types according to the classification of Tarantini et al. (1 if the coronary ostia were above the risk plane, 2a if the coronary ostia were below the risk plane with a valvular distance to the aorta (VTA) > 2 mm, 2b if the coronary ostia were below the risk plane with a VTA < 2 mm), and coronary access was assessed using PCI-concomitant angiography [12].

### 2.3. Statistical Analysis

All preoperative and intraoperative variables were first analyzed using univariate analysis (unpaired two-tailed *t* test, Mann–Whitney test, chi-square test or Fisher’s exact test when appropriate) to determine whether any single factor influenced mortality. Variables that achieved a *p* value less than 0.05 in the univariate analysis were examined using multivariate analysis with forward stepwise logistic regression to evaluate the independent risk factors for mortality. Survival curves were estimated at 1, 2, 5 and 8 years using the Kaplan–Meier method and compared using the log rank test. To adjust survival outcomes for possible influencing factors, Cox models were built and Hazard Ratios have been calculated.

## 3. Results

### 3.1. Preoperative Variables

The mean age was 82.2 ± 6.2 vs. 72.7 ± 9.7 years in the TAVI and SAVR populations, respectively. The mean EuroSCORE I, II and STS-PROM score, in TAVI and SAVR, were 15.9% vs. 7%, 5.2% vs. 2% and 4.5% vs. 2%, respectively. Extracardiac arteriopathy, chronic obstructive pulmonary disease (COPD), previous cardiac surgery, CAD, permanent AF, PM implantation, reduced ejection fraction (EF < 30% and/or 30% < EF < 50%), pulmonary hypertension (PAPs ≥ 60 mmHg), moderate/severe mitral regurgitation and a worse New York Heart Association (NYHA) class (III or IV) were more highly represented in the TAVI population. Smoking habits and bicuspid anatomy of the aortic valve were more common in the SAVR cohort. No differences in the aortic valve area (0.7 cm^2^ vs. 0.7 cm^2^ *P* ns) and mean aortic transvalvular gradient (48.5 mmHg vs. 49 mmHg *P* ns) were observed (Table 1).

### 3.2. Intraoperative Variables

Between 2010 and 2020, except for 2019 and 2020, aortic valve stenosis treatment increased. TAVI showed a steady growth, being the treatment of choice in 19% of cases in 2010 and reaching 54.4% in 2020. In the SAVR population, a bioprosthetic valve was implanted in 94.1% of patients with a mean age of 73.7 ± 8.4 years. The mean age of patients receiving a mechanical prosthesis (5.9%) was 56.1 ± 13.1 years. The mean extracorporeal circulation (ECC) time and mean cross-clamp time were 83 ± 21.8 and 60.6 ± 17.9, respectively. In the TAVI cohort, the most adopted approach was the transfemoral one (60.9%), followed by the transapical (24%), the transaortic (13.7%) and the transaxillary ones (1.4%).

The most implanted valve was the Edwards SAPIEN 3^®^ (*n* = 213, 48.7%), followed by the Edwards SAPIEN XT^®^ (*n* = 123, 28.2%), the Edwards SAPIEN 3 Ultra^®^ (*n* = 92, 21.1%) and the Edwards SAPIEN^®^ (*n* = 9, 2.1%). The THV 26 mm (*n* = 187, 42.8%) was the most frequently adopted size, then the THV 23 mm (*n* = 173, 39.6%), the THV 29 mm (*n* = 71, 16.2%) and the THV 20 mm (*n* = 6, 1.4%) (Table 2).

### 3.3. Postoperative Variables

The mean mechanical ventilation time, the mean intensive care unit (ICU) length of stay, the mean in-hospital stay, the need for blood transfusion and the postoperative AF incidence were statistically significantly lower in the TAVI cohort. Minor vascular complications and major bleeding were more represented in the TAVI population. No differences between the two cohorts concerning the incidence of postoperative kidney failure, transient ischemic attack (TIA), stroke and pacemaker (PM) implantation were observed. Comparing SAVR and transfemoral TAVI, a statistically significant difference in the in-hospital stay emerged, whereas no differences were observed comparing SAVR and non-transfemoral TAVI. Both SAVR and transfemoral TAVI showed a decrease in the in-hospital stay, according to the learning curve, from 12.8 and 9.6 days in the period 2010–2015 to 10.3 and 6.6 days in the period 2016–2020, respectively. TAVI was complicated by THV embolization once (0.23%), treated with THV stabilization in the abdominal aorta and a second THV implantation. Annulus injury occurred in 0.46% of cases and coronary obstruction (treated with PCI) in 0.69% of cases. In the TAVI population, a paravalvular leak more than moderate was detected in 2.1% of patients at discharge (Table 3).

### 3.4. Mortality

In the overall population, the 30-day mortality and in-hospital mortality were 1.2% (*n* = 18) and 1.6% (*n* = 24), respectively; they were 0.9% (*n* = 4) and 2.1% (*n* = 9) in the TAVI cohort and 1.3% (*n* = 14) and 1.4% in the SAVR cohort, with no statistically significant differences between the two procedures. In the bivariate analysis, higher risk scores (EuroSCORE 1, EuroSCORE 2 and STS-PROM) for End Stage Kidney Disease on dialysis, a worse NYHA class, mitral regurgitation more than moderate, a major blood procedural transfusion rate and major or minor bleeding were statistically significant.

### 3.5. All-Cause Mortality

**Figure 1:**
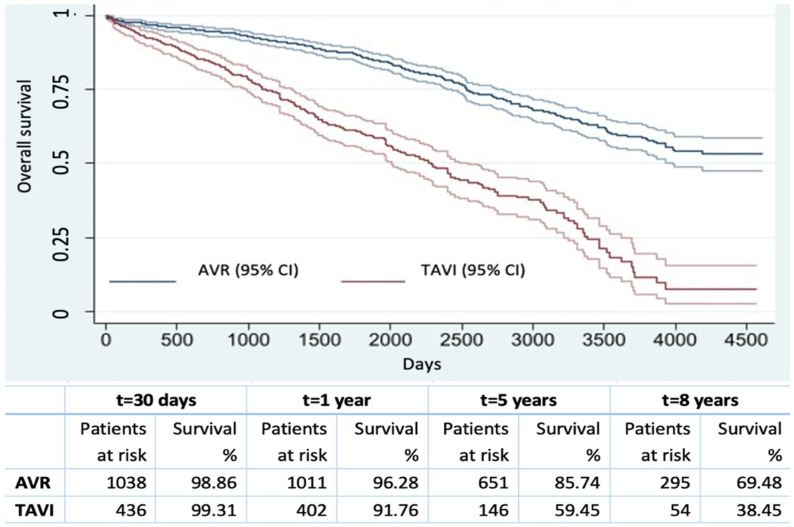
Overall survival after SAVR and TAVI.

**Figure 2:**
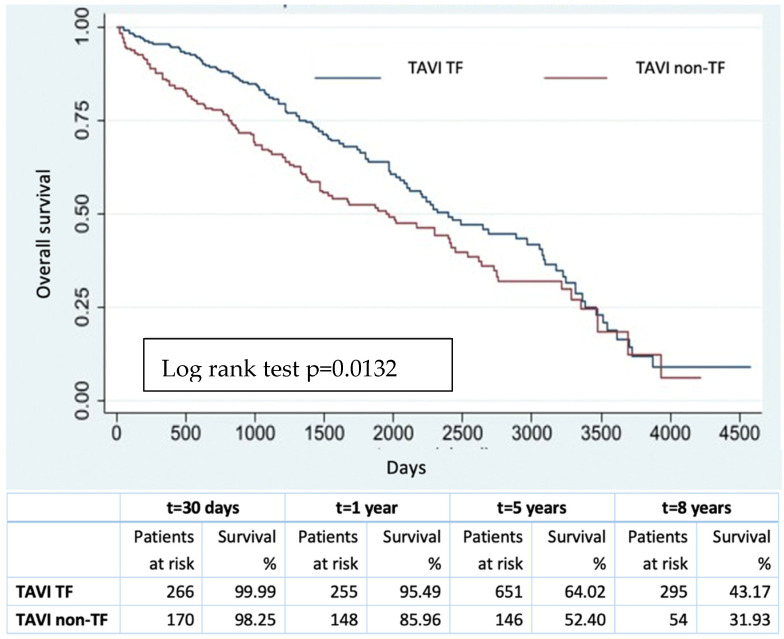
Overall survival after transfemoral TAVI and non-transfemoral TAVI.

The overall survival (all-cause mortality, classified into cardiac vs. non cardiac causes) at 30 days, 1 year, 5 and 8 years was 98.9%, 94.9%, 78.2% and 61.3%, respectively. Using the Cox model, age (HR 1.07, 95% CI 1.05–1.09 *p* < 0.001), COPD (HR 2.01, 95% CI 1.55–2.61 *p* < 0.001), an ejection fraction < 30% (HR 1.53, 95% CI 1.25–1.87 *p* < 0.001), preoperative atrial fibrillation (HR 2.32, 95% CI 1.85–2.93 *p* < 0.001), End Stage Kidney Disease on dialysis (HR 5.92, 95% CI 3.25–10.77 *p* < 0.001), TAVI as the procedure (HR 1.44, 95% CI 1.14–1.82 *p* = 0.003), blood transfusion (HR 1.75, 95% CI 1.46–2.11 *p* < 0.001), stroke/TIA (all-stroke VARC2 [13]) (HR 2.36, 95% CI 1.11–5.01 *p* = 0.026) and a longer in-hospital stay (HR 1.01, 95% CI 1–1.01 *p* = 0.030) were identified as independent predictors of long-term mortality. In the TAVI population, the adoption of an alternative approach was an independent predictor of all-cause mortality (HR 1.40, 95% CI 1.07–1.84, *p* = 0.014). The freedom from all-cause mortality at 30 days, 1 year, 5 and 8 years was 98.9%, 96.3%, 85.7% and 69.5%, and 99.3%, 91.8%, 59.5% and 38.5% in the SAVR and TAVI cohorts, respectively, with a 2046 (±1110) days/patient mean follow-up. The Kaplan–Meier survival estimates showed a statistically significant difference comparing the two procedures (log rank test *p* < 0.001) (Figure 1). In TAVI patients, the freedom from all-cause mortality was significatively different when comparing transfemoral TAVI vs. non-transfemoral TAVI (log rank test *p* = 0.013): at 30 days, 1 year, 5 and 8 years, it was 99.9% vs. 98.3%, 95.5% vs. 86%, 64% vs. 52.4% and 43.2 vs. 31.9%, respectively (Figure 2). Evaluating the long-term cardiac mortality, the Cox model showed age (HR 1.03, 95% CI 1.01–1.05 *p* = 0.002), COPD (HR 1.93, 95% CI 1.36–2.75 *p* < 0.001), preoperative AF (HR 1.41, 95% CI 1.07–1.85 *p* = 0.014), End Stage Kidney Disease on dialysis (HR 2.84, 95% CI 1.24–6.5 *p* = 0.014) and CAD (HR 1.33, 95% CI 1–1.75 *p* = 0.046) as independent predictors of mortality. Comparing the survival curves, the medians were 2800 days and 1600 days in terms of cardiac vs. non-cardiac mortality, respectively.

### 3.6. PCI

**Figure 3:**
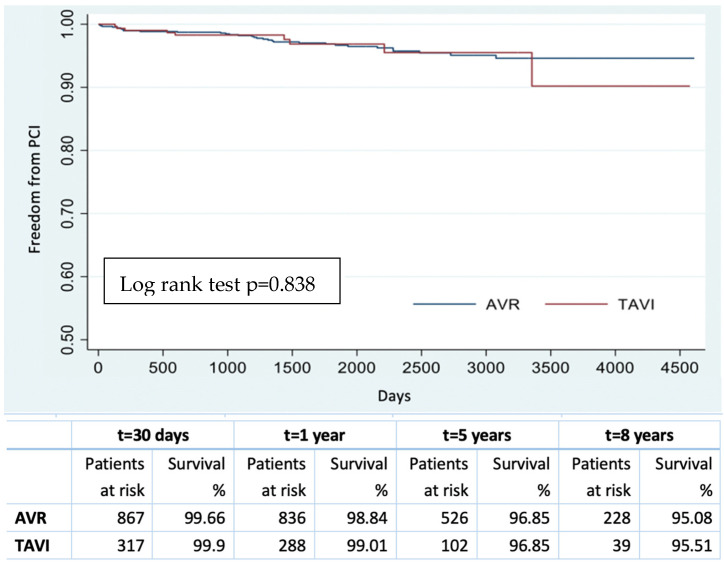
Freedom from PCI after SAVR and TAVI.

**Figure 4:**
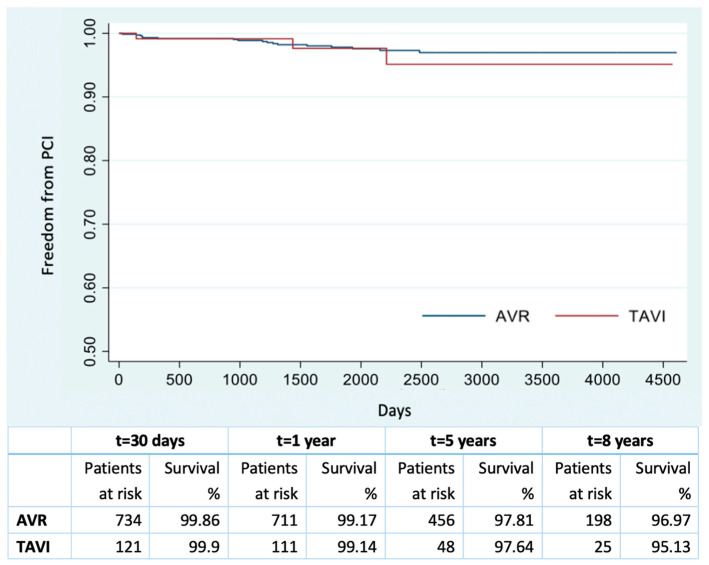
Freedom from PCI after SAVR and TAVI, excluding patients with a history of CAD.

The freedom from PCI in the overall population at 30 days, 1, 5 and 8 years was 99.75%, 98.88%, 96.85% and 95.14%, respectively, with a 1995 ± 1110 days/patient mean follow-up. The Cox model showed extracardiac arteriopathy (HR 3.08, 95% CI 1.60–5.90 *p* < 0.001) and previous PCI (HR 4.86, 95% CI 2.57–9.21 *p* < 0.001) as independent predictors of PCI after the procedure. In the SAVR population, the freedom from PCI at 30 days, 1, 5 and 8 years was 99.7%, 98.8%, 96.9% and 95.1%, respectively. In the TAVI patients, freedom from PCI at 30 days, 1, 5 and 8 years was 99.9%, 99%, 96.9% and 95.5%, respectively. Comparing the SAVR and TAVI freedom from the PCI curves, no statistically significant differences emerged (log rank test *p* = 0.838) (Figure 3). During follow-up, at least one percutaneous coronary intervention was performed in 41 patients (3.42%): 32 in SAVR and 9 in TAVI. Excluding patients with a history of CAD (27.7% overall, 15.5% in SAVR, 61.5% in TAVI, statistically significant difference *p* < 0.001), the freedom from the PCI curves was not significantly different (log rank test *p* = 0.659). Moreover, the freedom from PCI at 30 days, 1 year, 5 and 8 years was 99.86%, 99.17%, 97.81%, 96.97% and 99.9%, 99.14%, 97.64%, 95.13% in the SAVR and TAVI populations, respectively (Figure 4). At least one percutaneous coronary intervention was performed in 17 SAVR patients (2.3%, 17/734), while, in the TAVI population, it occurred in 3 patients (2.4%, 3/121) (log rank test *p* = 0.312).

Nine cases of PCI after TAVI were reported: six in patients with a history of CAD and three in patients without CAD. In the CAD group, three patients underwent PCI with a SAPIEN XT^®^, two with a SAPIEN 3^®^ and one with a SAPIEN 3 Ultra^®^. The coronary ostia of all patients treated using a SAPIEN XT^®^ and SAPIEN 3^®^ Ultra were above the risk plane (type 1), while with a SAPIEN 3^®^, the coronary ostia were below the risk plane with a VTA < 2 mm (type 2b). Considering patients who underwent PCI without a history of CAD, two were implanted with a SAPIEN XT^®^ and one with a SAPIEN 3^®^. In this clinical subset of patients, the coronary ostia of patients treated using a SAPIEN XT^®^ were above the risk plane (type 1), whereas with a SAPIEN 3^®^, the coronary ostia were below the risk plane with a VTA < 2 mm (type 2b). All the clinical and procedural details of the PCI are summarized in Table 4.

## 4. Discussion

Over the last decade, TAVI has been established as a form of treatment for severe aortic valve stenosis and is now recommended by European and American guidelines [9,10]. At the beginning, it was performed only in inoperable or high-risk patients [1,2] but severe trials confirmed its effectiveness even in moderate- and low-risk patients [5,6,14,15], who usually are younger. Actually, a non-negligible question is the lifetime management of these patients, mainly related to the long-term THV durability and coronary re-access. It is mandatory to study the epidemiology of the TAVI population and to analyze the PCI predictors highlighted. All these aspects are essential to tailor the best operative path for patients in the heart team strategy. The aim of our study was to analyze these themes in mainly inoperable and high-risk patients undergoing TAVI, with Edwards balloon-expandable devices, in the decade 2010–2020, comparing the findings coming from this real-world clinical setting with others from trials and real-world experience.

The TAVI and SAVR populations of the study reflect the typical real-world clinical practice procedural trend of severe aortic valve stenosis in the second decade of the 21st century [16]. TAVI was the treatment of choice mainly for inoperable or high-risk patients [2], different from the actual screening process population, which includes even younger and intermediate-risk patients [9]. TAVI patients were older and with a heavy comorbidity burden (extracardiac arteriopathy, COPD, CAD, worst NYHA class, previous cardiac surgery) in comparison with the SAVR ones. Preoperative atrial fibrillation and PM implantation, which are both age-related conditions, were also more common in the TAVI cohort. The TAVI outcomes were comparable with the SAVR ones, probably due to the restrictive and guideline-driven strategy adopted by the local heart team.

**Figure 5:**
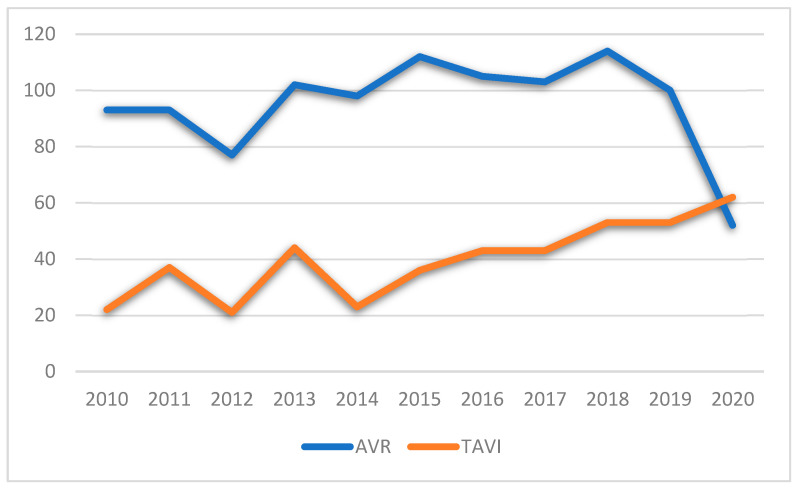
Trend of SAVR and TAVI during the 2010–2020 decade in Hesperia Hospital Modena, Italy.

Intraoperative variables analysis showed an annual increasing number of applications of TAVI (except for in 2019–2020 due to the COVID-19 pandemic), similar to what happened all over Europe [17] (Figure 5). During the COVID-19 pandemic, a TAVI-driven strategy for severe aortic valve stenosis was adopted, with an increased TAVI/SAVR ratio, explainable considering the delay or cancellation of elective cardiac surgery (like SAVR) and the lower impact of TAVI on in-hospital stay [18,19].

**Figure 6:**
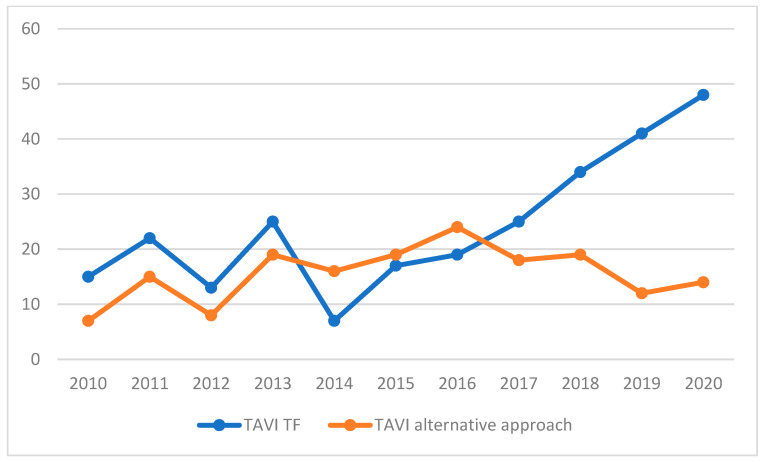
Trend of transfemoral TAVI and non-transfemoral TAVI during the 2010–2020 decade in Hesperia Hospital Modena, Italy.

**Figure 7:**
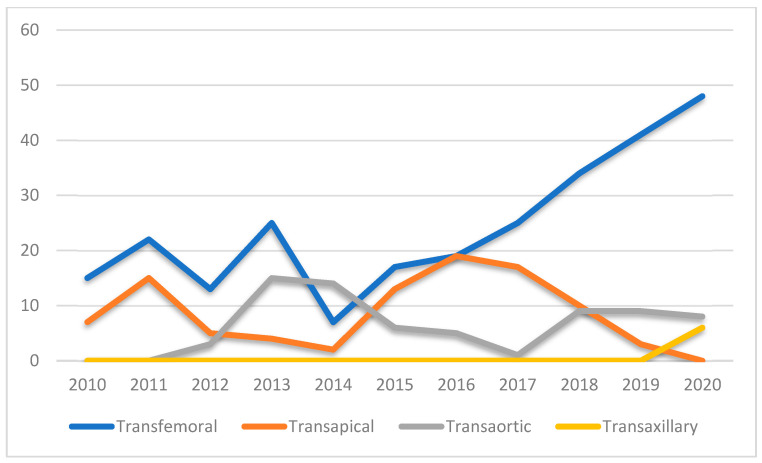
Trend of TAVI approaches during the 2010–2020 decade in Hesperia Hospital Modena, Italy.

The transfemoral TAVI growth reflects the Edwards device evolution, characterized by a reduction in the sheath diameter (from 24 Fr with Cribier-Edwards^®^ to 14 Fr with SAPIEN 3^®^) [20]. However, the cardiac surgeon’s involvement in the heart team’s process allowed the adoption of transfemoral strategy as non-obligatory, resulting in 20% of patients receiving an alternative approach in 2020. This result is comparable with the 15% of patients reported in the literature [21,22], and could explain the low incidence of major vascular complications (2.1%) (Figure 6 and Figure 7).

The overall 30-day mortality, 1.2%, was lower in comparison with the PARTNER 1A (4%), 2A (3.9%) and United States SAVR-TAVI registry (3.5%) 30-day mortality [2,14,23]. However, the reported 30-day mortality for each procedure (0.9% in SAVR vs. 1.3% in TAVI) was similar to other figures from real-world experience. Di Eusanio et al., the UK National Database and Takeji et al. reported a 2.2%, 1.9% and 1.3% 30-day mortality for SAVR [24,25,26], whereas the 30-day mortality for TAVI was 1.8% in the UK TAVI registry and 3.17% in the Nationwide Inpatient Sample registry [27,28].

Both SAVR and TAVI reached 78.2% overall survival at 5 years. Age, COPD, EF < 30%, permanent preoperative AF, End Stage Kidney Disease on dialysis, blood transfusion, stroke/TIA and in-hospital stay emerged as long-term mortality independent predictors. All of these factors are well known in the literature for their predictiveness [24,25,29,30,31,32,33,34,35,36] (Table 5).

In spite of this, the role of a longer in-hospital stay in predicting long-term mortality was not previously identified as an independent predictor for long-term mortality. A bicuspid aortic valve morphology seems a protective factor against all-cause mortality. Holmgren, comparing tricuspid and bicuspid SAVR, showed the same result [37]. In TAVI patients, the opinion that the mid-term outcomes in bicuspid aortic valve patients would be non-inferior when compared with tricuspid aortic valve patients is spreading [38]. Zhou et al., in a population with a bicuspid aortic valve as the etiology in 43% of cases, reported 87.1% 3-year survival vs. 79.5% for patients with a tricuspid anatomy [39]. An alternative approach in TAVI emerged as an all-cause mortality independent predictor in our population, like in the PARTNER trials and in the UK TAVI registry (transapical approach and transaortic approach (HR 1.74, 95% CI 1.43–2.11 *p* < 0.001; HR 1.55, 95% CI 1.13–2.14 *p* = 0.01)) [40,41]. Focusing on the procedure and its influence on long-term survival, except for 30-day mortality (lower in the TAVI cohort), a longer survival in SAVR patients was detected and TAVI was identified as an all-cause mortality independent predictor (HR 1.44, 95% CI 1.14–1.82 *p* = 0.003). The impact of SAVR on short-term mortality is well known, in trials (PARTNER 1A, 6.5% and 3.4% 30-day mortality after SAVR and TAVI, respectively) and in real-world practice (United States Transcatheter and Surgical Aortic Valve Replacement Registry, 30-day mortality of 3.7% in SAVR and 2.4% in TAVI) [2,23]. However, an early survival trend inversion was detected, with the 1-year survival still better in SAVR patients in comparison with TAVI patients (96.3% vs. 91.8%), while looking at PARTNER 1A (which focuses on high-risk patients, similarly to our population), survival inversion happens after 5 years [42]. The trend detected in our study was probably related to the TAVI patients’ profiles, who were older with a higher comorbidity burden. In fact, looking at the 5-year survival (40.5%) and taking into account PARTNER 1A (32.2%) our study showed an excellent performance for TAVI [42].

Analyzing the cardiac vs. non-cardiac mortality medians (2800 days vs. 1600 days), it seems that non-cardiac causes of death have a stronger impact on survival once aortic valve stenosis is removed using TAVI or SAVR.

The Cox models revealed CAD (previous PCI or CABG and/or previous myocardial injury and/or recent myocardial injury) as a long-term predictor of cardiac mortality. This correlation is well known in the literature, both for TAVI (Kaihara et al. (HR 5.32, 95% CI 1.55–18.21 *p* = 0.008), Dewey et al. and Ludman et al.) [27,43,44] and SAVR (Beach et al.) [45]. In terms of SAVR, the impact of CAD on mortality is so relevant that it is included in the most frequently applied risk scores [46,47].

### PCI

In SAVR patients, concomitant coronary artery disease represents the most common and important comorbidity able to influence the procedural outcome. More than 30% of overall patients suffer from coronary artery disease and it is even more diffuse in patients aged 70 years or more, up to 50% [48]. The need for myocardial revascularization after SAVR was analyzed by Celik et al. in 420 patients (mean age 56.9 ± 15.5 years), who underwent isolated SAVR from 1978 to 2015, with a 17.2-year mean follow-up, showing a revascularization incidence of 6.9% at 20 years (24 patients), most of them treated for PCI (64%). An overall incidence of revascularization after SAVR of 0.5%, 0.5%, 2.2%, 4.1%, 5.3% and 6.9% at 30 days, 1 year, 5, 10, 15 and 20 years, respectively, was reported. Previous coronary revascularization (PCI in 75% of cases) was identified as an independent predictor of coronary revascularization after SAVR (HR 6.6, 95% CI 2.6–17.1, *p* < 0.001) [49].

In TAVI patients, concomitant coronary artery disease is reported in 40–75% of cases (59% in our population), and the adopted definitions’ discrepancy could justify this wide range [50]. Concomitant coronary artery disease with aortic valve stenosis in TAVI patients represents a negative prognostic factor, especially in patients with a higher Syntax Score (≥22) before PCI, or with a higher residual Syntax Score (>8) after PCI [51,52]. The best PCI timing have not yet been identified by European guidelines, while American guidelines recommend treating a specific subgroup of patients. Epicardial vessel stenosis > 70% in the proximal segments or left main stenosis > 50%, whether the PCI procedure’s risk overcomes the potential benefits or not, should undergo PCI before TAVI, while stenoses at the mid or distal parts of the coronary vasculature or stenoses with small areas of ischemia should be treated after TAVI [53]. Our strategy was to perform PCI within 60 days of TAVI in all patients with a higher Syntax Score (≥22) before PCI, or with a higher residual Syntax Score (>8) after PCI. Nevertheless, up to now, the need to treat coronary artery disease in a patient undergoing TAVI remains a matter of debate. Kotronias et al. performed a meta-analysis of nine observational studies, taking 4000 TAVI patients with concomitant coronary artery disease untreated or treated with PCI (before or after the procedure) and evaluating the all-cause mortality and the mortality due to cardiovascular causes at 30 days and 1 year after TAVI. At 30 days, PCI was associated with a higher all-cause mortality and with a higher incidence of major vascular complications, whereas, after 1 year, no remarkable differences in all-cause mortality and mortality due to cardiovascular causes were observed [54]. The ACTIVATION trial was the first randomized trial of PCI versus no PCI prior to TAVI in patients with severe AS and significant coronary disease, and was designed to prove the noninferiority of PCI prior to TAVI strategy. There was no evidence of a difference in the primary endpoint of death or rehospitalization at 1 year between patients who did or did not undergo PCI prior to TAVI. Furthermore, in the PCI-treated cohort, the incidence of bleeding events was higher [55]. In our real-world experience, the vascular complications and bleeding incidence in patients undergoing PCI prior to TAVI were not different from patients who did not undergo PCI.

Concerning PCI after TAVI, only a few data are available. The review by Weferling et al. reported a coronarography incidence of 2.5–3.5% in TAVI patients, with PCI performed in 27–55% of cases [56]. Nai Fovino et al., in a cohort of 912 patients with a 2.1-year mean follow-up, reported a 5.3% coronarography incidence, due to acute coronary syndrome in 35% of cases, with PCI performed in 26 patients (54%). The incidence of PCI after TAVI was 2.8%, with a younger age, a previous PCI and/or a previous CABG being independent predictors of coronarography [57]. The SOURCE 3 Registry, including 1936 patients treated using a SAPIEN 3^®^, showed a 3.5% incidence of coronarography during a 3-year follow up, with indicators represented by stable angina (36.8%), NSTEMI (26.5%) and STEMI (11.8%); PCI was performed in 69% of cases, with a 2.4% overall incidence of PCI [58]. In our population, the PCI overall incidence after SAVR was 3.6%, in a 5.4-year mean follow-up, with a rate of 0.3%, 1.2%, 3.1% and 4.9% at 30 days, 1 year, 5 and 8 years, respectively. Celik, with a 17.2-year mean follow-up, reported a 0.5%, 0.5%, 2.2%, 4.1% and 6.9% incidence of revascularization (including CABG) at 30 days, 1 year, 5, 10 and 20 years, showing a trend similar to our experience [49]. Instead, in TAVI patients, PCI was performed in 2.8% of cases, with a rate of 0.1%, 1%, 3.1%, 4.5% at 30 days, 1 year, 5 and 8 years, respectively. Analog data are available in the literature, even if a shorter follow-up is usually considered. Nai Fovino et al. reported a 2.8% PCI incidence after TAVI in a 2.1-year follow-up, and Tarantini and colleagues reported a 2.4% PCI incidence at 3 years of follow-up [57,58]. A possible pitfall is represented by coronary re-access. Edwards balloon-expandable THV technology is characterized by a low profile frame, even if, after device evolution (Edwards SAPIEN XT^®^, then Edwards SAPIEN 3^®^ and Edwards SAPIEN 3 Ultra^®^), the profile frame was raised, but this was compensated for with a 38% increase in the open cell area in the upper part of the frame [59]. Considering the latest-generation Edwards SAPIEN 3 Ultra^®^, the 40%-raised outer skirt suggests more difficult coronary re-access, even considering the emerging habit of performing high implantations, aiming to reduce cardiac conduction disorders [60]. Faroux et al., comparing the Edwards SAPIEN 3^®^ THV with the SAPIEN XT^®^, confirmed a higher coronary ostia closure, but without compromising PCI or coronarography feasibility [61]. In our experience, coronary re-access was performed both above (type 1) and through the frame upper cells (type 2b), confirming the operative strategies proposed by Tarantini et al. according to the anatomical relationship between the THV risk plane and the coronary ostia takeoff [62]. During follow-up, only successful cases of PCI were detected, making it impossible to analyze the causes of failed PCI, including the reasons for eventual impossible coronary re-access.

Excluding patients with a clinical history of CAD (angina, previous PCI, previous CABG) before the procedure, in order to select a cardiac-ischemia-free population at the time of the TAVI procedure, the overall PCI incidence after TAVI and SAVR was 2.4% and 2.3%, respectively, with a 6.8-year mean follow-up. Despite the age of the population being high, especially in TAVI (>80 years), the revascularization incidence after TAVI (2.4%) is not significant. This may only be partly related to the mean old age of the TAVI population. However, this aspect could be remarkable if we consider the shift in TAVI for moderate/low-risk patients, who usually are younger, with a need for PCI that could be higher, and the coronary access feasibility, especially using high-profile THVs, should be evaluated. The exclusion of patients with severe CAD and recent PCI treatment from intermediate- and low-risk TAVI trials holds an open question on the real need for PCI after TAVI and collides with the ongoing trend of offering TAVI even to a less comorbidity-burdened population. Recently, Tarantini et al. focused on these unresolved questions, analyzing that ongoing trials, designed to assess the correlation between PCI and TAVI (TAVI PCI Trial, COMPLETE TAVR trial), could help to overcome the actual doubts, providing new guidelines on the treatment of coronary artery disease [63]. Outcoming data from these studies will give an useful orientation, but only real-world practice will be able to clear define this “gray area”.

According to the Cox model, previous PCI (HR 4.86, 95% CI 2.57–9.21 *p* < 0.001) emerged as an independent predictor of PCI after TAVI, in our real-world experience. Similar results were reported by Celik after SAVR and Nai Fovino et al. after TAVI, detecting previous PCI and previous CABG as independent predictors of coronarography after the procedure, with a 2.8% overall PCI-after-TAVI incidence [49,57]. Extracardiac arteriopathy, as defined by the European risk score system [46], was found to be an independent predictor of a subsequent PCI in our experience. This variable, which includes four diseases (*claudicatio intermittens*, as a result of peripheral artery disease and/or carotid occlusion or >50% stenosis and/or previous surgery on the abdominal aorta, limb arteries or carotids and/or amputation for arterial disease), is notoriously associated with an increased in-hospital mortality after coronary interventions, such as PCI and CABG, and it can predict other complications, especially the need for PCI independent from other comorbidities [64,65,66,67].

## 5. Limitations of the Study

The study presents all the intrinsic limitations of observational retrospective studies, as well as discrepancy in the data collection, changes in definitions of comorbidities and loss of patients during the follow-up. However, in 2010–2020, all data were collected by the same physician, the comorbidity definitions did not change and patients were registered in the same database (RERIC). Moreover, using the Italian ANPR database, no loss of patients in terms of mortality during follow-up occurred. Other outcomes were evaluated only for Emilia Romagna residents (*n* = 1196, 80.5% of overall population) using the hospital discharge records (SDO) system and linking the new hospital admissions characterized by the diagnosis codes of cardiovascular disease and cardiovascular interventions with the index procedure. Over the entire period of the study, the choice of TAVI or AVR, made by the local heart team, was influenced by the outcoming data from PARTNER trials 1A and 1B (1, 2). Inoperable and high-risk patients were selected for TAVI, while AVR was performed in all others, introducing a bias of selection, which can be observed considering the remarkable heterogeneity of our two populations. All TAVI patients were treated using balloon-expandable THVs, allowing comparison with the procedural outcomes from PARTNER trials. Nevertheless, we could not translate our outcomes in the case of self-expandable THV usage. The adopted method of data collection did not permit us to find cases of PCI being required but not feasible, leading to a potential underestimation of the need for PCI.

## 6. Conclusions

Both the AVR and TAVI results were extremely satisfactory for all primary outcomes, especially survival, even in a mid-term follow-up, and showed a low complication incidence. TAVI in real-world experience confirmed the findings of trials concerning inoperable and high-risk patients. The low coronary revascularization after TAVI using balloon-expandable devices, detected in patients without CAD, could allay concerns about coronary re-access, despite this aspect having to be evaluated in a younger TAVI population. The coronary revascularization predictors need to be deeply evaluated, in order to define the tailored lifetime management of patients, especially considering the actual trend of performing TAVI in younger patients, who have a greater chance of developing CAD in the future. Ultimately, the timing of CAD treatment has not yet been defined. In our experience the PCI-prior-to-TAVI strategy was not associated with a higher incidence of bleeding and vascular complications, as recently reported in the literature.

## Figures and Tables

**Table 1:** Preoperative variables.

Preoperative Variables	SAVR	TAVI	*p* Value
EuroSCORE 1 (Logistic)	7.0 ± 4.9	15.9 ± 9.4	<0.001
EuroSCORE 2	2.0 ± 1.7	5.2 ± 4.0	<0.001
STS SCORE	2.0 ± 1.2	4.5 ± 2.8	<0.001
Age (mean)	72.7 ± 9.7	82.2 ± 6.2	<0.001
Age (median)	75.0	83.2	<0.001
Female	498 (47.5%)	235 (53.8%)	0.027
BMI	27.5 ± 5.0	27.3 ± 5.0	0.4292
BSA	1.8 ± 0.2	1.7 ± 0.2	<0.001
Diabetes	194 (18.5%)	101 (23.1%)	0.042
Hypertension	993 (94.7%)	433 (99.1%)	<0.001
Hypercolesterolemia	659 (62.8%)	296 (67.7%)	0.072
Dialysis	8 (0.8%)	6 (1.4%)	0.267
Preoperative creatinine	1.1 ± 3.6	1.5 ± 7.5	0.145
Stroke	21 (2%)	19 (4.4%)	
TIA	32 (3.1%)	23 (5.3%)	0.004
Smoking habits	544 (51.9%)	167 (38.2%)	<0.001
COPD	60 (5.7%)	76 (17.4%)	<0.001
Extracardiac arteriopathy	152 (14.5%)	118 (27.0%)	<0.001
Bicuspid anatomy of aortic valve	182 (17.4%)	7 (1.6%)	<0.001
Previous cardiac surgery	29 (2.8%)	61 (14.0%)	<0.001
Previous CABG	6 (0.6%)	49 (11.2%)	<0.001
Previous surgery of cardiac valves	18 (1.7%)	14 (3.2%)	0.072
Previous PCI	111 (10.6%)	216 (49.4%)	<0.001
Previous myocardial injury	37 (3.5%)	63 (14.4%)	<0.001
Recent myocardial injury (EuroSCORE)	18 (1.7%)	14 (3.2%)	0.072
CAD	150 (14.3%)	260 (59.5%)	<0.001
Preoperative AF	100 (9.5%)	83 (19.0%)	<0.001
NYHA III/IV class	304 (29.0%)	383 (87.6%)	<0.001
EF < 30%	185 (17.6%)	113 (25.9%)	<0.001
Aortic regurgitation	119 (11.3%)	82 (18.8%)	<0.001
Mitral regurgitation	52 (5.0%)	76 (17.4%)	<0.001
Tricuspid regurgitation	27 (2.6%)	69 (15.8%)	<0.001
Pulmonary hypertension	11 (1.1%)	22 (5.0%)	<0.001
PM dependency	13 (1.2%)	30 (6.9%)	<0.001
Liver cirrhosis	5 (0.5%)	3 (0.7%)	0.700
Active cancer	11 (1.1%)	5 (1.1%)	0.871
Senile degeneration	1009 (96.2%)	431 (98.6%)	0.019
Degeneration of tricuspid valve	33 (3.1%)	3 (0.7%)	<0.001
Degeneration of bicuspid valve	7 (0.7%)	3 (0.7%)	<0.001
Aortic valve area	0.7 cm^2^	0.7 cm^2^	ns
Mean aortic transvalvular gradient	48.5 mmHg	49 mmHg	ns

BMI: Body Mass Index; BSA: Body Surface Area; COPD: Chronic Obstructive Pulmonary Disease; CABG: Coronary Artery Bypass Graft; PCI: Percutaneous Coronary Intervention; CAD: Coronary Artery Disease; AF: Atrial Fibrillation; NYHA: New York Heart Association; EF: Ejection Fraction; PM: Pacemaker; ns: not statistically significant.

**Table 2:** Procedural variables.

Procedural Variables	SAVR	TAVI
Mechanical valve	62 (5.9%)	
Cross-clamp time	60.3 ± 17.9	
ECC time	83.0 ± 21.8	
Edwards SAPIEN^®^		9 (2.1%)
Edwards SAPIEN XT^®^		123 (28.2%)
Edwards SAPIEN 3^®^		213 (48.7%)
Edwards SAPIEN 3 ULTRA^®^		92 (21.1%)
Transfemoral approach		266 (60.9%)
Transapical approach		105 (24%)
Transaortic approach		60 (13.7%)
Transaxillary approach		6 (1.4%)
20 mm THV		6 (1.4%)
23 mm THV		173 (39.6%)
26 mm THV		187 (42.8%)
29 mm THV		71 (16.2%)

ECC: Extracorporeal Circulation; THV: Transcatheter Heart Valve.

**Table 3:** Postoperative variables.

Postoperative Variables	SAVR	TAVI	*p* Value
Blood transfusion	529 (50.4%)	166 (38.0%)	<0.001
Mechanical ventilation time (hours)	14 ± 62.4	13.1 ± 58.8	<0.001
ICU length of stay (days)	2.2 ± 6.6	1.7 ± 3.5	0.129
Acute renal failure stage 1	25 (2.4%)	9 (2.1%)	0.751
Acute renal failure stage 2	5 (0.5%)	2 (0.5%)	
Acute renal failure stage 3	11 (1.1%)	2 (0.5%)	
Minor bleeding	5 (0.5%)	4 (0.9%)	0.017
Major bleeding	17 (1.6%)	17 (3.9%)	
Minor vascular complications	0 (0%)	31 (7.1%)	<0.001
Major vascular complications	0 (0%)	9 (2.1%)	
Percutaneous vascular complications	0 (0%)	13 (3.0%)	
Stroke	8 (0.8%)	2 (0.5%)	0.732
TIA	4 (0.4%)	1 (0.2%)	0.999
Postoperative AF	332 (31.7%)	12 (2.8%)	<0.001
PM implantation	24 (2.3%)	10 (2.3%)	0.999
In-hospital length of stay (mean)	11.6 ± 11.9	9.6 ± 12.0	0.004
In-hospital length of stay (median)	9	7	<0.001
Embolization		1 (0.23%)	
Annular injury		2 (0.46%)	
Coronary obstruction		3 (0.69%)	
Perivalvular leak more than moderate		9 (2.1%)	

ICU: Intensive Care Unit; TIA: Transient Ischemic Attack.

**Table 4:** Patients undergoing PCI after TAVI with and without a history of CAD.

Patient	Type of Device	Time Lapse Between TAVI and PCI (Years)	Type of Risk Plane in CT Scan	Coronary Access
1	SAPIEN XT^®^	5	1	Above the frame
2	SAPIEN XT^®^	4	1	Above the frame
3	SAPIEN XT^®^	1.8	1	Above the frame
4	SAPIEN 3^®^	0.4	2b	Through the open cells
5	SAPIEN 3 ULTRA^®^	1.6	1	Above the frame
6	SAPIEN 3^®^	0.7	2b	Through the open cells
7	SAPIEN XT^®^	3.6	1	Above the frame
8	SAPIEN XT^®^	6	1	Above the frame
9	SAPIEN 3^®^	0.5	2b	Through the open cells

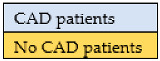
.

**Table 5:** Independent predictors of all-cause mortality.

Independent Predictors	Our Experience	SAVR	TAVI
Age	HR 1.07, 95% CI 1.05–1.09	HR 1.03, 95% CI 1.02–1.04	HR 1.15, 95% CI 1.01–1.30
		Jahangiri et al. [25]	Attinger-Toller et al. [29]
COPD	HR 2.01, 95% CI 1.55–2.61	OR = 2.6, 95% CI 1.6–15.7	HR 1.84, 95% CI 1.08–3.13
		Di Eusanio et al. [24]	Mok et al. [30]
Permanent AF	HR 2.32, 95% CI 1.85–2.93	HR 2.24, 95% CI 1.79–2.79	HR 3.1, 95% CI 1.05–9.19
		Farag et al. [32]	Eftychiou et al. [31]
EF < 30%	HR 1.53, 95% CI 1.25–1.87	HR 1.7, 95% CI 1.2–2.5	HR 2.86, 95% CI 1.4–5.8
		Di Eusanio et al. [24]	Puls et al. [33]
ESKD on dialysis	HR 5.92, 95% CI 3.25–10.77	HR 9.8, 95% CI 2.4–47.5	HR 1.18, 95% CI 1.06–1.33
		Di Eusanio et al. [24]	Nuis et al. [34]
Blood transfusion	HR 1.75, 95% CI 1.46–2.11	HR 1.19, 95% CI 1.05–1.33	HR 3.1, 95% CI 1.5–6.7
		Vlot et al. [35]	Nuis et al. [34]
Intraoperative TIA or stroke (All-stroke VARC-2)	HR 2.36, 95% CI 1.11–5.01	HR 5.2, 95% CI 3.07–8.80 Kodali et al. [36]	HR 2.47, 95% CI 1.42–4.30 Kodali et al. [36]

*p* value considered were all statistically significant (*p* < 0.001).

## Data Availability

The data are stored in the RERIC Hesperia Hospital registry and in the Emilia Romagna healthcare system (concerning the hospital discharge records), but both are unavailable due to privacy.
